# *Nigella sativa* monophosphoryl lipid A nanoliposome: a promising antibiotic alternative and immunomodulator to control virulent pandemic drug-resistant *Salmonella pullorum* infection in broiler chicks

**DOI:** 10.1186/s12917-025-04473-w

**Published:** 2025-03-03

**Authors:** Adel Attia M. Ahmad, Elsayed Alsaied Masoud Hussien, Alaa A. A. M. Elian, Mohamed Abdelmoneim, A. Ali, Ahmed E. Abdelhamid, Gamal A. Elmowalid

**Affiliations:** 1 Microbiology Department, Faculty of Veterinary Medicine, Zagazig University, Sharqiah, Egypt; 2 Animal Health Research Institute, Agriculture Research Center, Dokki, Giza, Egypt; 3 Pathology Department, Faculty of Veterinary Medicine, Zagazig University, Sharqiah, Egypt; 4 Polymers and Pigments Department, National Research Center, Giza, Dokki, Egypt

**Keywords:** *Nigella sativa* MPLA nanoliposomes, *Salmonella pullorum*, Broiler chicks, Pro-inflammatory cytokines, IgA, Mucin-2

## Abstract

**Background:**

*Salmonella enterica* serovar Pullorum, the causative agent of pullorum disease, is one cause of the economic losses in the global poultry industry. Vaccination and antibiotics are still the most effective methods of controlling Salmonella, even though the vaccine contains the causative agent, and the antibiotic therapy has limited efficacy. We provide a novel immunostimulator and antibiotic substitute to protect against and avoid *Salmonella pullorum* (SP) infection.

**Methods:**

*Nigella sativa*-purified oil (NS) and monophosphoryl lipid A (MPLA) were formulated as nanoliposomal compounds (NS-MPLA). Their protective and immunomodulatory efficacies were experimentally tested orally in broiler chicks against challenge with virulent pandemic drug-resistant SP. Four chick groups were utilized: control; NS-MPLA-supplemented; SP-challenged; and SP-challenged, then NS-MPLA-treated. Clinical signs, organ gross pathology, colony-forming counts, and tissue histopathological alterations were investigated. The relative fold-changes in the expression of *IL-1β, IL-4, IL-17*, *IL-22, TLR-4, INF-γ, IgA,* and *MUC2* genes were evaluated.

**Results:**

The SP-challenged chicks showed notable symptoms and extensive pathological lesions in their internal organs. The bacteria colonized the challenged chicks' livers and continued to shed in their feces for 5–6 days. A minor amount of immune cell tissue trafficking was noted. The NS-MPLA-treated chicks displayed opposing patterns after being challenged with SP. They exhibited mild clinical signs with modest gross pathology in the internal organs. After 3–4 days, the liver and the fecal droppings were cleared of SP. Significant heterophilic aggregation, lymphocytic infiltration, and lymphoid follicle enlargement were observed. Additionally, chicks challenged with SP and then NS-MPLA-treated showed a 5- to tenfold increase in immune-related cytokines, immunoglobulin A, and mucosal relative gene expression folds compared to the SP-challenged non-NS-MPLA-treated, which showed a sharp decline in *IL-4* and *IL-22* and a minor rise in the rest of the tested gene relative expressions. Chicks given NS-MPLA supplementation showed a significant upregulation of these genes compared to the control group.

**Conclusion:**

In this first report on poultry, it is possible to draw the conclusion that NS-MPLA supplementation in SP-infected chicks boosts immunity and provides protection. It promoted bacterial clearance and tissue repair and stimulated the expression of genes linked to immunity and the mucosal surface. These findings suggest the potential application of NS-MPLA in salmonella control programs as an antibiotic substitute or in immunization strategies.

**Supplementary Information:**

The online version contains supplementary material available at 10.1186/s12917-025-04473-w.

## Introduction

*Salmonella enterica* biovars Pullorum and Gallinarum are responsible for chicken typhoid and pullorum disease, respectively. Pullorum disease, which causes severe symptoms and mortality in its acute stage, is common in poultry [1]. Because the causal agent spreads from the internal organs over months, the chronically infected flock may acquire latent and persistent sickness, even though the adult chickens may not exhibit any signs [2]. Antibiotic treatment of flocks with carrier states is never advised. Purchasing birds free of *Salmonella enterica* Pullorum, protecting flocks from salmonella infection, and serologic screening of causal agents are the cornerstones of routine breeding flock control. Due to Salmonella's multidrug resistance in developing countries, the majority of Salmonella isolates in those countries are currently at least resistant to three or more antimicrobials [3, 4].

Vaccinating chickens against *Salmonella pullorum* (abbreviated SP) infections is another efficient way to increase resistance to infection exposure and decrease bacterial shedding. To provide enough defenses against severe SP infections, the attenuated SP ghost vaccine was developed [5]. According to the European Food Safety Authority, EFSA, vaccination may or may not be a suitable substitute, depending on the objectives of the control program (eradication or reduction), the type of poultry, the stage of production, the prevalence of Salmonella, the serovars being targeted, the detection techniques, and the cost–benefit analysis [6]. In poultry farming around the world, using antibiotics to prevent disease and promote growth is standard procedure. Antibiotics such as tetracycline, bacitracin, tylosin, and salinomycin are used in industrial poultry production in North America; meanwhile, virginiamycin was often employed [7]. However, food safety and public health may be at risk due to the rise of antibiotic-resistant bacterial strains and their human transmission. On January 1, 2017, the Food and Drug Administration (FDA) prevented the use of antibiotics that are significant for human medicine and prohibited them from being used to promote growth or feed efficiency in cows, pigs, chickens, turkeys, or other food animals [8]. As a result, it is critical to investigate natural compounds as antibiotic substitutes for the poultry industry.

In poultry, *Nigella sativa* (*N. sativa*) seed and oil have been utilized as food supplement agents, immunomodulatory agents, and nutritional supplements [9–12]. According to the data, *N. sativa* improved performance and survivability [13], prevented gastric lesions with a stable gastric mucosal redox state [14], lowered intestinal lesion score and caecal morphology in infected broilers [15, 16], and eliminated the additional expenses of antibiotics and other vaccinal preparations with an easy-to-apply administration route. The majority of these studies, however, used *N. sativa* as crude seed or oil dietary supplements, which were found to have low bioavailability and necessitate high dosages, longer periods of time, and continuous administration in order to achieve the desired effect.

According to extensive research, thymoquinone (TQ), the active ingredient in *N. sativa*, and its essential oil have a variety of therapeutic benefits, such as antibacterial and immunomodulatory activities. The poor absorption of TQ, hydrophobicity, and *N. sativa* oil diminished their therapeutic effects. Nanoformulations, or nanoparticles, were therefore suggested. Numerous formulations of nanoparticles were created and delivered by nasal, parenteral, ophthalmic, oral, and other routes. The encapsulation of *N. sativa* oil, or TQ, into nanoparticle forms improved solubility and absorption, preserved the molecularly dispersed form in the upper gastrointestinal system, and increased protection against harsh, unfavorable conditions through oral ingestion [17–20].

*Salmonella enterica* endotoxin was used to create monophosphoryl lipid A (MPLA), a toll-like receptor-4 agonist that is a detoxified version of lipid A. The United States Food and Drug Administration authorized MPLA as a safe immunostimulatory adjuvant, and it is currently being used in clinical trials and new commercial vaccines. When administered to mice, it produced significant levels of salivary, plasma, and vaginal immunoglobulin A (IgA)-specific antibodies. It was made with oral immunizing antigen (*Streptococcus mutans* crude glucosyl transferase) into liposomal membranes (MPLA adjuvant formulation) [21]. It produced a mucosal and systemic cellular immunity that was faster, stronger, and more durable [22–25]. In addition to activating macrophages, MPLA also improved cytokines, lymphokines, and T- and B-cell interactions. MPLA was previously employed as a secure non-aluminum vaccine adjuvant. A Th1 or a combined Th1 and Th2-type response was observed in most studies that focused on the quality of the immune response induced in both clinical and non-clinical grade variants of MPLA [26, 27].

The existing chicken immunization and/or antibiotic control program has some drawbacks, as reported EFSA [7, 28, 29]. Among these drawbacks are the possibility of microbial resistance to antibiotics, interference with monitoring protocols, and potential human infection with the vaccine-contained microbial strains, in addition to the inability to completely stop microbial shedding or infection with other *Salmonellae*. This program also requires strong immune protection against non-typhoidal *Salmonellae* gut colonization, which raises the cost of the vaccines used. Therefore, the development of safe and effective alternatives is required. Recently, the construction of plant-origin potent immunomodulatory compounds against poultry pathogens has gained more attention. In this study, natural, purified *N. sativa* essential oil (NS) with a bacterial derivative, MPLA, was formulated in nanoliposomal form (NS-MPLA) and experimentally tested in broiler chicks. To the best of our knowledge, this is the first report on the use of NS-MPLA in an experimental study as an antibiotic alternative and immunomodulatory agent against virulent pandemic drug-resistant (PDR) SP infection in poultry.

## Materials and methods

### *N. sativa* oil and monophosphoryl lipid A (MPLA)

Purified *N. sativa* essential oil, NS (cold-pressed seeds), was purchased from Haraz Company for Natural Herbal Products in Egypt. Detoxified monophosphoryl lipid A (MPLA), produced from *Salmonella minnesota* Re 595 (Re mutant), was purchased from Sigma Aldrich in St. Louis, MI, USA. Both MPLA and NS were formulated to develop *N. sativa*-purified oil-detoxified monophosphoryl lipid A (NS-MPLA) nanoliposomes. Here, "NS-MPLA" refers to both the developed *N. sativa* essential oil and the detoxified monophosphoryl lipid A nanoliposomes.

### The NS*-*MPLA nanoliposomes development

The procedures of [30, 31] were applied to develop NS-MPLA nanoliposomes encapsulated in chitosan. Using magnetic stirring, a half gram of low molecular weight chitosan (Mwt: 100–300 KDa, Across Co Ltd Company, UK) was dissolved in 50 ml of aqueous acetic acid (2%) to generate solution (A). Then 0.25 g of soy lecithin was dissolved in 25 ml of deionized water and agitated for one hour at 60 ˚C. Consequently, 0.25 g of pure essential oil (NS) from *N. sativa* was added to the soy lecithin solution dropwise while stirring. After dissolving 1.0 mg of MPLA in 1.0 ml of dimethyl sulphoxide (DMSO), the MPLA was added to the soy lecithin and NS mixture and agitated dropwise for 30 min to generate solution (B). The aqueous phase of the chitosan solution was homogenized at 15,000 rpm for 15 min after solution B was added drop by drop to solution A to ensure a well-dispersed nanoemulsion of liposomes and hydrophobic molecules. To create a soy lecithin liposome with a nanochitosan shell that compromises both the monophosphoryl detoxified lipid A (MPLA) and *N. sativa* purified oil (NS), 0.34 g of sodium tripolyphosphate was dissolved in 25 ml of double-deionized water and added to the prepared mixture drop by drop while being gently stirred. The mixture was agitated for 30 min after the sodium tripolyphosphate solution was added, and then it was removed and stored at 4 °C until it was characterized. It was then utilized either alone or post challenging broiler broiler chicks with SP.

### Characterizations of NS-MPLA nanoliposomes

#### Transmission Electron Microscope (TEM)

The produced NS-MPLA nanoliposomes were inspected using a transmission electron microscope (HR-TEM, JEOLJEM-2100) to determine their size and distribution. The freshly synthesized suspension of nanoliposomes was placed onto the carbon-coated mesh grid for testing, allowed to air dry completely, and then inspected under the TEM.

#### Dynamic light scattering (DLS)

The Zeta Sizer (Nano-ZS, Malvern Ltd., UK) was used to determine the size and zeta potential of the prepared NS-MPLA. To guarantee evenly distributed nanoparticles in the aqueous medium, the suspension was subjected to a one-minute sonication. The suspended particles' Brownian motion produced the scattered beams seen in the DLS measurements of the nanoliposomes.

### Isolation and identification of the challenging SP strains

From private broiler farms in the Sharqiah governorate, thirty freshly deceased broilers were brought to the Bacteriology Diagnostic Unit at the Faculty of Veterinary Medicine, Zagazig University, Egypt. The chicks' livers and spleens were aseptically dissected to get them ready for bacterial isolation [32]. A loopful of the processed specimens was inoculated into Rappaport–Vassiliadis broth and then cultivated for 48 h at 40 °C. The established bacterial growth was subcultured onto XLD agar media following the incubation period. API20-E strips were used to detect tiny, transparent, suspicious red colonies [33]. Using these two specific F and R primers, 5′-CAGCGTTTAAGCTGCCAGACCCAG GCC-3′ and 5′-TTCGCGACG AAT TTA AAG AGAGCG AAG-3′, respectively, the *flhB* gene in S. Pullorum/Gallinarum was amplified by conventional PCR in compliance with the methodology of [34]. The SP isolates were further characterized by tests for ornithine decarboxylase, rhamnose consumption, glucose utilization, and absence of motility in indicator motility media. Prior to being employed in the chick challenge and undergoing testing for antibiotic susceptibility, the isolates that were confirmed to be SP were kept at −20 °C.

### Susceptibility testing of SP against NS and antimicrobials

As previously reported [35], the disc diffusion method was used to assess the identified SP isolates' susceptibility to both NS and antimicrobials. In brief, a disc of Whatman filter paper coated with 25 µl of NS soaked in 10% DMSO was adhered to the surface of Muller Hinton agar medium (MHA, Oxoid, UK) after half of McFarland’s density standard of freshly prepared SP suspension (1.0 × 10^8^ CFU/mL) had been placed over it. Similarly, the antimicrobial susceptibility of the identified SP strains was determined using a panel of nine antimicrobial discs (Oxoid, UK) that represented different antimicrobial groups and included ampicillin, amoxicillin, doxycycline, colistin, cefotaxime, chloramphenicol, azithromycin, sulphamethoxazole/trimethoprim, and amoxicillin/sodium clavulanate. The SP strains that were verified to be resistant to NS and the nine antimicrobial-tested (pandemic drug-resistant, PDR) strains were used in the chick experimental challenge.

### Experimental design, chicks' management, colonization breakage, and SP challenge

This investigation was experimentally designed using one hundred and sixty one-day-old Ross chicks that were Salmonellae-free and purchased from Tiba Company. The chicks were acclimated in a clean environment for 14 days, receiving water and a basic diet devoid of food supplements daily. No antibiotics or other antimicrobials were provided throughout the acclimatization phase. They were further confirmed to be Salmonellae-free on the fifteenth day of their lives after enriching the liver, spleen, and fecal droppings in tetrathionate broth (Neogene, MI, USA) at 37 °C for six hours, then inoculating 10 µl of the broth onto Rappaport–Vassiliadis agar and incubating at 41 °C for twenty-four hours. Ten milligrams of neomycin, five milligrams of vancomycin, ten milligrams of metronidazole, and three milligrams of fluconazole in drinking water were then administered to the chicks for 12 h over the course of two days in order to induce intestinal bacterial colonization breakage and the experimental infection [36]. The chicks were then kept in their cages for an additional twenty-four hours before being randomly distributed to the following four experimental groups:

#### Group I (negative control group) 

The 40 chicks in this group received two divided doses of PBS, separated by 6 h, orally gavaged at a dose of 5.0 ml per chick.

#### Group II (NS-MPLA-supplied group)

 This group's 40 chicks received only oral NS-MPLA (2.5 mg NS oil and 10 ng MPLA for each chick). Two doses (1.0 ml per chick, spaced six hours apart) of the NS-MPLA supplement were administered.

#### Group III (SP-challenged group)

A freshly prepared PDR-SP (10^8^ CFU/ml) suspension was used to induce infection in the chicks (*N* = 40). The PDR-SP challenge bacteria were administered orally to the chicks in two consecutive doses (1.0 ml per chick, separated by 6 h).

#### Group IV (SP-challenged, followed by NS-MPLA treatment group)

The 40 chicks of this group were given the challenging SP in the same way as in group III, and then, each bird was given 1.0 ml of NS-MPLA (2.5 mg NS oil and 10 ng MPL per chick) orally in two divided doses, separated by six hours.

Clinical symptoms and gross pathological lesions in internal organs were observed after the chicks were challenged with and/or given NS-MPLA alone as a supplement or as a treatment after the SP challenge. To calculate CFU counts, bacterial isolation was performed on liver and fecal droppings samples from the dying or recently deceased chicks. In order to analyze histological changes in the SP-challenged, NS-MPLA-treated, and NS-MPLA-supplemented chicks following challenge, liver, spleen, and caecum specimens were also collected. To examine IgA, immune-related cytokines, and mucosal-regulating genes, samples were extracted from the caecal tonsils of the four experimental groups.

### NS-MPLA nanoliposomes protective and immunomodulatory activities against SP experimental challenge

#### Clinical signs

Following the chicks' challenges with the virulent PDR-SP, the clinical symptoms, internal organ lesions, and mortalities were assessed in the four chick groups for two weeks.

#### The SP-CFU counts in the chicks' liver tissues and fecal droppings

The bacterial counts per gram were calculated by centrifuging the liver and/or fecal dropping samples from the dissected chicks at a low speed (500 rpm) after they had been aseptically homogenized in PBS and sterile sand. The following is an estimate of the SP-CFU counts in the processed samples: Before inoculating Salmonella Shigella agar or Rappaport–Vassiliadis agar plates, 10 µL of the sample supernatants were ten-fold serially diluted in sterile PBS. The plates were inspected, and the SP-CFU counts were calculated using the previously described method [32] following a 48-h incubation period at 41 °C.

#### Histopathological alterations

The liver, caecum, and spleen specimens were kept in 10% neutral buffered formalin after being dissected from chicks that were SP-challenged and subsequently NS-MPLA-treated, as well as chicks that were SP-challenged. Following the standard procedure, 5 µm tissue slices were sectioned, paraffin-prepared, and stained with hematoxylin and eosin in order to perform microscopic investigations [37].

#### Quantitative real-time PCR (RT-qPCR)

##### RNA extraction and cDNA synthesis

Following the mentioned references [38–40], the primers listed in Table 1 were used to determine the expression of the immune-related cytokines, immunoglobulin A, and mucosal genes in the cecal tonsils on days 1, 2, 3, and 7 of the four tested chick groups. Following the manufacturer's instructions, total RNA was extracted from the cecal tissues using the Trizol TM method (Invitrogen, USA). The amount and quality of extracted RNA were assessed at 260 and 280 nm using a UV–visible spectrophotometer. To rule out any genomic DNA contamination, the DNase treatment was carried out using the DNase1 kit (Sigma, USA). cDNA synthesis was performed using the Thermo Scientific Revert Aid First Strand cDNA Synthesis Kit (Lithuania), and every sample had the same RNA concentration (1.5 μg/μl) in each of the cecal tissue samples that were collected from the tested chicks.

**Table 1:** Primers used in real-time quantitative PCR

Target gene	Primer sequence	Reference
β-actin	F-5'-CATCAGGGAGTCATGGTCGGCA-3’ R-5'GCATACAGGTCTTTACGGATGTCC-3'	Gene bank JQ086312
IL-17	F-5’-ATGGGAAGGTGATACGGC-3’ R-5’ GATGGGCACGGAGTTGA-3	Gene bank AM773756
IL-22	F-5'-GCACCTACACCTTGGCTGAA-3’ R-5'TGTAGCAGCGGTTGTTCTCC-3’	GenBank: AJ617782.1
IFN-γ	F-5'-GTGAAGAAGGTGAAAGATATCATGGA-3' R-5'-GCTTTGCGCTGGATTCTCA-3'	[38] [38]
IL-1β	F-5'-CAGCCTCAGCGAAGAGACCTT-3' R-5'-CACTGTGGTGTGCTCAGAATCC-3'	
IL-4	F-5'-AATGACATCCAGGGAGAGGTTTC-3' R-5'-AGGCTTTGCATAAGAGCTCAGTTT-3'	[39]
TLR-4	F-5'-TGTGCTGGAAAAAGCTGCAC-3' R-5'-AGCTTTGTCTCACGGGTCAC-3'	Gene bank, KP410249.1
MUC-2	F: 5'-CAGCACCAACTTCTCAGTTCC-3' R:5'-TCTGCAGCCACACATTCTTT-3'	[40] [40]
IgA	F: 5'-ACCACGGCTCTGACTGTACC-3' R: 5'-CGATGGTCTCCTTCACATCA-3'	

*F *forward, *R* reverse, *β-actin* cytoskeletal structural protein, *MUC-2* mucin-2, *IgA* immunoglobulin A, *INF-γ* interferon- γ, *IL-1β* interleukin 1β, *IL-4* interleukin 4, *TLR-4* toll-like receptor-4

##### Quantitation of mRNA expression

Target chick genes were measured for mRNA expression levels using quantitative real-time PCR, or qRT-PCR. As directed by the manufacturer, the QuantiTect SYBR Green fluorescent dye one-step RT-PCR kit (Qiagen) and the Mx3000P real-time qPCR device (Stratagene, La Jolla, CA) were used to measure the level of gene expression. The studied gene expression was normalized by using β-actin as an internal control. The 2 − ΔΔCT comparative approach was used to determine the relative amount of the expressed gene mRNA [41]. ΔΔCT was used to depict the difference between the CT measurements for the mRNA level of each tissue and the CT measurements for the mRNA level of the reference gene. ΔCT (sample control) is equal to CT (target gene) minus mean CT (β-Actin).

## Statistical analysis

The mean ± standard error of CFU counts and immune-related and mucosal-regulating genes was analyzed by analysis of variance in the different experimental groups. The data was analyzed by one-way analysis of variance (ANOVA).

## Results

### NS-MPLA morphology and distribution

**Fig. 1:**
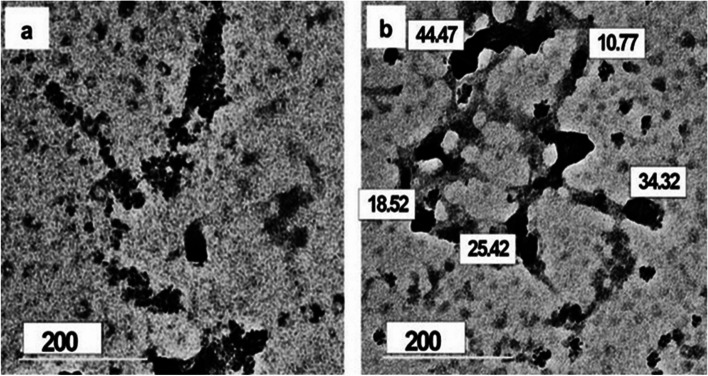
** a** and **b**: TEM images of the synthesized nanoliposomes at two different locations. The nanoliposomes are dark gray and semispherical in shape (**a**) with some dark black aggregates (**a**). The sizes of the nanoliposomes 10-45 nm (**b**)

As seen in Figs. 1a and b, TEM was used to assess the morphology of the developed nanocapsules. The resulting nanoparticles were dark and semi-spherical (Fig. 1a). Within the capsules, the encapsulated MPLA is located in a dark area. The particles, which ranged in size from 10 to 45 nm on average, exhibited some aggregation and an interconnected structure, as is typical of NS-MPLA chitosan nanoparticles (Fig. 1b).

### NS-MPLA nanoliposomes zeta potential

As shown in Supplementary Data 1a and b, DLS was used to analyze the synthesized NS-MPLA's size and charges. The nanoliposomes had a size of about 28.5 nm, making them comparatively monodisperse (supplementary data 1a). A big peak with a positive value of 8.24 mV and a little peak with a negative value of −5.36 mV were observed in the zeta potential (supplementary data 1b).

### *S. pullorum* (SP) identification, and antimicrobial and NS susceptibility

**Fig. 2:**
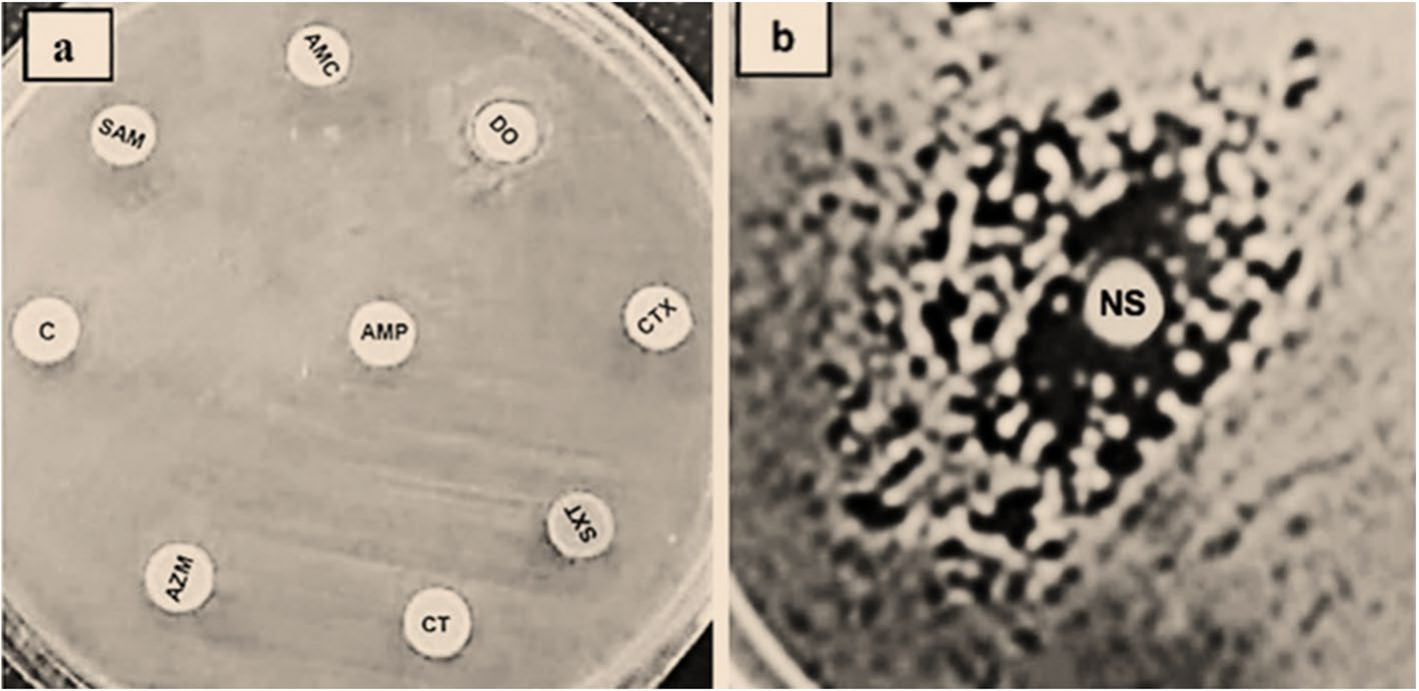
**a** and **b**: Antimicrobials and *N. sativa *purified oil susceptibilities of the *S. Pullorum* challenging strain. In the disc diffusion, the tested SP strain showed pandemic resistance to the nine tested antimicrobials (**a**) and to the purified *N. sativa* oil (**b**). CT: colistin; AZM: azithromycin; SXT: sulphamethoxazole/trimethoprime; AMP: ampicillin; C: chloramphenicol; SAM: ampicillin/sulbactam; CTX: cefotaxime; DO: doxycycline; AMC: amoxycillin/sodium clavulanate

Six strains were confirmed as SP following culture, biochemical, and genetic identification. Only one SP strain exhibited resistance to both NS and the tested antimicrobials (PDR strain) when tested against the nine antimicrobials and NS (Figs. 2a and b). The remaining five strains exhibited resistance to the NS and to at least four of the tested antimicrobials (multidrug-resistant). The PDR-SP strain was used for the chick challenges (groups III and IV).

### CFU counts in the SP-challenged chicks and those treated with NS-MPLA post-SP challenge

For 15 days, the number of SP-CFU in the chicks' livers and fecal droppings was counted daily. Prolonged SP fecal shedding was seen in the chicks in group III that were challenged with SP. SP remained in the feces for 4–5 days and in the liver for 3–4 days, as seen in Table 2. The SP challenged chicks that were subsequently treated with NS-MPLA (group IV) had a high level of bacteria (6.6 ± 0.0.45 × 10^8^) at day 1, which was completely eliminated from the liver tissues and fecal droppings by day 4 and day 5, respectively, but the bacteria remained in the fecal droppings of the SP-challenged chicks, group III (Table 2 and Supplementary Data 2a-d). By the sixth to the fifteenth day, neither the liver tissues nor the feces showed any signs of bacterial shedding in both group III and group IV (Table 2).

**Table 2:** *S. pullorum* colony-forming unit counts in the chicks fecal droppings and liver tissues of SP-challenged and the SP-challenged, followed by NS-MPLA treatment at different time intervals

Time	Fecal droppings		Liver tissues	
	**SP-challenged**	**SP-challenged, then NS-MPLA-treated**	**SP-challenged**	**NS-MPLA treated** **post-SP challenge**
**1st d**	1.3 ± 0.5 × 10^8^	6.6 ± 0.0.45 × 10^8^	0.0	0.0
**2nd d**	1.5 ± 0.3 × 10^8^	3.7 ± 0.25 × 10^8^	0.0	0.0
**3rd d**	4.7 ± 0.4 × 10^6^	4.0 ± 0.6 × 10^8^	3.9 ± 0.4 × 10^4^	3.0 ± 0.5 × 10^2^
**4th d**	6.0 ± 0.3 × 10^3^	8.0 ± 0.75 × 10^6^	2.5 ± 0.35 × 10^4^	0.0
**5th d**	1.0 ± 0.25 × 10^3^	0.0	0.0	0.0
**6th −15**^**th**^**d**	0.0			

*d* day, *SP* *S. pullorum,* *NS-MPLA* *N. sativa*-purified oil-monophosphoryl lipid A nanoliposomes

#### The pathological alterations

##### Clinical symptoms in the SP-challenged chick groups

**Fig. 3:**
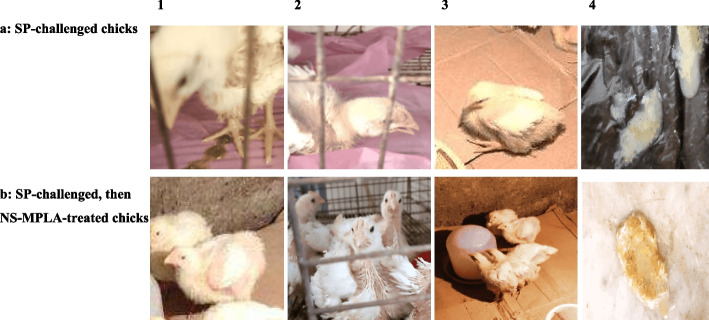
** a** and **b**: The clinical signs in the SP-challenged chicks and those NS-MPLA treated post-SP challenge. The SP-challenged chicks (**a**) exhibited a dropped head (1), stretched neck (2), and an inability to move (3) with watery fecal droppings (4), while the SP-challenged, then NS-MPLA-treated chicks (**b**) showed the opposite view

The experimental challenge of the chicks (groups III and IV) was conducted using the confirmed SP-PDR strain, and the clinical signs were observed for 15 days following the SP challenge and NS-MPLA administration. As seen in Fig. 3a, the SP-challenged chicks in group III exhibited clinical symptoms on the second and third days after the challenge, which included depression, dullness, drooping wings, neck stretching, anorexia, unwillingness to move, and white fecal droppings. Growing weakness, anorexia, ruffled feathers, falling of wings, and lowering of the head were all observed in the second week post-challenge. As shown in Fig. 3b, the opposite scenario was observed in the SP-challenged, then NS-MPLA-treated chicks (Group IV), with non-significant clinical symptoms at day 3–4 post-challenge (2–3 days post-NS-MPLA treatment). The chicks in the same group (IV) showed mild clinical symptoms starting in the third day and continued for only one day post-treatment with NS-MPLA. The chicks, who were given NS-MPLA alone (group II), were healthy and physically fit; there were no deaths or health issues reported (data not shown).

#### Gross pathology of the chicks' internal organs

##### Internal organ lesions

**Fig. 4:**
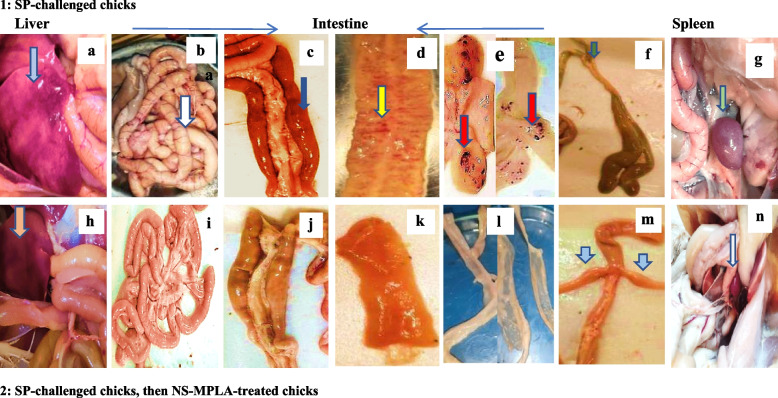
(**a**-**n**): Internal organ pathological lesions in chicks SP-challenged (1) and SP-challenged, followed by NS-MPLA treatment (2). The SP-challenged chicks (**a**–**g**) showed signs of congested hemorrhagic liver with mottled appearance (**a**, light blue arrow), congested distended bowel (**b**, white arrow), dark intestinal contents (**c**, dark blue arrow), petechial hemorrhage (**d**, yellow arrow) or patchy bloody spots (**e**, red arrows), small diminished cecal tonsils (**f**, green arrow), and an enlarged congested spleen (**g**, light green arrow). Following the SP challenge and subsequent NS-MPLA treatment (**h**-**n**), the chicks displayed modest intestinal changes (**i**-**l**), cecal tonsil enlargement (**m**, light blue arrows), and an apparently normal liver and spleen (**h** and **n**, light brown and light gray arrows, respectively)

As shown in Fig. 4 (a-g), prominent internal organ prominent lesions (intestine, liver, and spleen) were observed in the chicks that were challenged with the SP. Dark fecal contents, gas-filled swelling, congestion, and bleeding patches were all seen in the gut. Additionally, the cecal tonsils were diminished. Furthermore, the liver displayed mottling, and the spleen was enlarged and congested. In contrast, the NS-MPLA-treated chicken post SP-challenge showed mild internal organ lesions with enlargement in their cecal tonsils (Figs. 4 (h-n).

#### The immunohistopathological view of the internal organs

##### Liver, caecum, and spleen immunohistopathological alterations

**a. Liver**

**Fig. 5:**
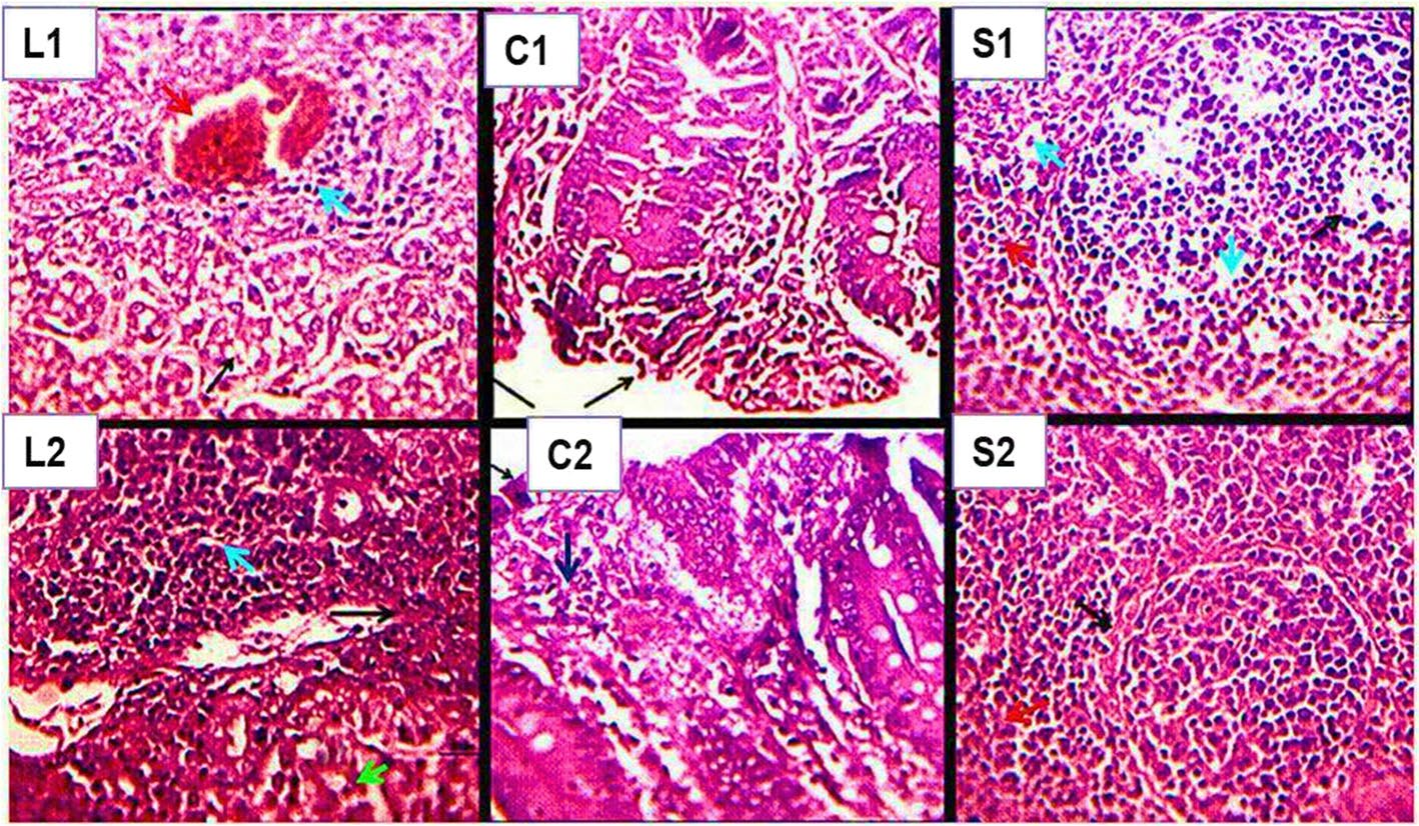
Histological changes in the liver (L), caecum (C), and spleen (S) of chicks challenged with SP and those challenged with SP and then treated with NS-MPLA. The sections from the liver (L1, L2), spleen (S1, S2), and caecum (C1, C2) were included. The SP-challenged chicks provided the C1, L1, and S1 sections, while the SP-challenged chicks that subsequently received NS-MPLA treatment provided the C2, L2, and S2 sections. The NS-MPLA-treated chicks' hepatic tissues (L2) displayed intense portal heterophilic and lymphocytic aggregates (light blue arrow) with hyperplastic Kupffer cells and heterophilic cell trafficking (black and green arrows), whereas the hepatic tissues of those SP-challenged chicks (L1) displayed mild portal leukocyte aggregates (light blue arrow) and parenchymal swelling with clogged blood vessels (red arrow). In contrast, the cecal tissues of the chicks treated with NS-MPLA after the challenge (C2) showed lymphocytic infiltration (black and light blue arrows), a normal mucosal surface (C2), and identifiable heterophilic cells, mostly mononuclear cells. A denuded surface (black arrow) and mucosal injury with modest heterophilic and lymphocytic infiltration (green and light blue arrows) were observed in the chicks challenged with SP (C1). The white pulps of the SP-challenged and NS-MPLA-treated chicks (S2) showed intensely determined hyperplasia and histiocytic proliferations (black and red arrows); the low splenic white pulps of the SP-challenged chicks (S1) showed proliferative reticulo-endothelial cells (histiocytes), and mild heterophilic infiltration was seen in the depleted splenic white pulps (light blue and red arrows) of SP-challenged chicks (S1). H&E X 400 arrows

There were noticeable differences in the liver tissues of the studied chicks, according to the histopathology sections that were examined. Significant changes were seen in the liver of the SP-challenged chicks (group III), including hepatic parenchyma expansion and blood vessel congestion along with portal leukocyte aggregation, mostly of mononuclear cells (Fig. 5L1, Table 3). The liver tissues of the chicks that were given NS-MPLA after being challenged with SP (group IV) displayed hyperplastic Kupffer cell enlargement along with intense portal heterophilic lymphocytic aggregates and parenchyma that appeared to be normal. No hepatic degenerations were found (Fig. 5L2, Table 3). At the same time, Kupffer cell hyperplasia and seemingly normal portal heterophilic and/or lymphoid plasmocytic aggregates were seen in the chicks supplemented with NS-MPLA (group II) (Table 3).

**Table 3:** Pathological alterations in the chicks liver of NS-MPLA-supplied, SP-challenged, and those SP-challenged, then NS-MPLA-treated at different time intervals

Organ	Pathologic alterations	NS-MPLA supplied (Group II)				SP-challenged (Group III)				NS-MPLA treated post-SP challenge (Group IV)				
		1d	2d	3d	4d	1d	5d	9d	13d	1d	2d	5d	9d	13d
**Liver**	**Hydrobic degeneration**	-	-	-	-	+	+	++	-	++	+	-	-	-
	**Lympho plasmo-cytic infiltration**	+++	+++	+++	+	+	+	++	+	++	++	++	+++	++
	**Periportal Heterophilic aggregations**	**-**	**-**	+++	**+**	**-**	**-**	**++**	**-**	**+++**	**++**	**++**	**+++**	**++**
	**Kupffer cell hypertrophy**	**+**	**++**	**+++**	**+**	**++**	**+**	**++**	**-**	**+++**	**++**	**+**	**+++**	**++**
	**Focal biliray proliferation**	**-**	**-**	**-**	**-**	**-**	**-**	**-**	**-**	**+**	**+**	**+**	**+**	**-**

*d* day, *SP S. pullorum, NS-MPL* *N. sativa- *monophosphoryl lipid A nanoliposomes

**b. Caecum**

The cecal tissues of the SP-challenged (group III) and treated (group IV) chicks showed clear differences in the histopathological sections that were analyzed. Figure 5C1 and Table 4 demonstrate the presence of cecal tissue injury in the SP-challenged chicks. Thickened lamina propria, a denuded enterocyte surface, slight lymphocytic and heterophilic infiltration, submucosal edema, and moderate hyperplasia with proliferative intestinal glands were all examples of this injury. Beginning on the first day of infection and continuing for nine days, the SP-challenged chicks showed signs of lymphoid tissue loss (Table 4). The cecal tissues of group IV treated with NS-MPLA after the SP-challenge showed villi epithelial lining reconstitution, with notable heterophilic and lymphocytic infiltration in the mucosa and intense heterophilic infiltration. Table 4 and Fig. 5C2 indicate that the cecal tissues of the group IV treated with NS-MPLA after the SP-challenge showed reconstitution of the villi epithelial lining, an intense hyperplasia of lymphoid follicles with an intact epithelial surface, and significant heterophilic and lymphocytic infiltration in the mucosa and intestinal glands. These findings persisted for at least 13 days. The chicks were supplemented with NS-MPLA. Along with proliferative intestinal glands, the chicks supplemented with NS-MPLA (group II) displayed a notable hyperplasia of submucosal lymphoid follicles in the cecal wall. This was associated with mucosal lymphocytic and/or heterophilic infiltration (Table 4).

**Table 4:** Pathological alterations in the chicks caecum of NS-MPLA-supplied, SP-challenged, and those SP-challenged, then NS-MPLA-treated at different time intervals

Cecal alterations	NS-MPLA supplied (Group II)				SP-challenged (Group III)				NS-MPLA treated post-SP challenge (Group IV)				
	1d	2d	3d	4d	1d	5d	9d	13d	1d	2d	5d	9d	13d
**Mucosal damage**	+	-	-	-	++	++	++	+	+	+	+	+	+
**lymphocyte infiltration**	++	++	++	++	+	+	++	++	+	++	+++	+++	+++
**Mucosal heterophilic infiltration**	--	-	-	++	++	+	+	-	-	++	++	+++	++
**Size of lymphoid follicles**	++	++	+++	++	-●	-●	-●	++	+	+++	++	+++	++
Glandular epithelial proliferation and regeneration	++	++	++	++	+	+	++	++	+	++	+++	+++	+

*d *day, *SP S. pullorum,* *NS-MPL* *N. sativa- *monophosphoryl lipid A nanoliposomes. -●: depleted

**c. Spleen**

According to histopathology-inspected sections shown in Fig. 5S1 and Table 5, the splenic white pulps of the SP-challenged chicks (group III) showed proliferative reticuloendothelial cells (histiocytes). There was little heterophilic penetration in the red pulps. In contrast, the splenic tissues of group IV chicks treated with NS-MPLA after the SP challenge showed a significant hyperplasia of white pulps and histiocytic proliferations. Heterophils and macrophages were also significantly infiltrated into the red and white pulp (Fig. 5S2 and Table 5). With proliferating reticuloendothelial cells, the chicks in Group II, who were given only NS-MPLA supplementation, displayed hyperplasia in the majority of the white pulps and mild heterophilic infiltration mostly in the red pulps (Table 5). In the liver, caecum, and spleen of the SP-challenged, NS-MPLA-treated (group IV), and supplemented (group II) chicks, immune cell trafficking was observed beginning on the second and third days and continued for at least 13 days (Tables 3, 4, and 5). This trafficking included lymphocytic and heterophilic infiltrations of internal tissues as well as lymphoid follicle enlargement. The same two groups also showed signs of glandular gland renewal and proliferation. The liver, caecum, and spleen all showed mildly immunological cell trafficking, particularly in the initial two days after the SP challenge, which lasted for just nine days (Tables 3, 4, and 5).

**Table 5:** Pathological alterations in the chicks splenic tissues of NS-MPLA-supplied, SP-challenged, and those SP-challenged, then NS-MPLA-treated at different time intervals

Organ	Pathologic alteration	NS-MPLA-supplied (Group II)				SP-challenged (Group III)				NS-MPLA treated post-SP challenge (Group IV)				
		1d	2d	3d	4d	1d	5d	9d	13d	1d	2d	5d	9d	13d
**Spleen**	**Lymphoid hyperplasia of white pulps**	++	+++	+++	++	+	+	++	+	-	+++	+++	+++	+++
	**Heterophils around red pulps**	++	-	+++	-	-	-	-	+	-	++	++	+++	+++
	**Histocytic proliferation**	+++	+++	+++	++	+++	+	++	+	++	+++	+++	+++	+++
	**Lymphocytic infiltration in red pulps**	-	-	-	-	-	-	-	-	+	+++	++	++	++

*d* day, *SP S. pullorum, NS-MPL* *N. sativa- *monophosphoryl lipid A nanoliposomes

### The expression of the immune-related cytokines, immunoglobulin A and mucosal genes

**Fig. 6:**
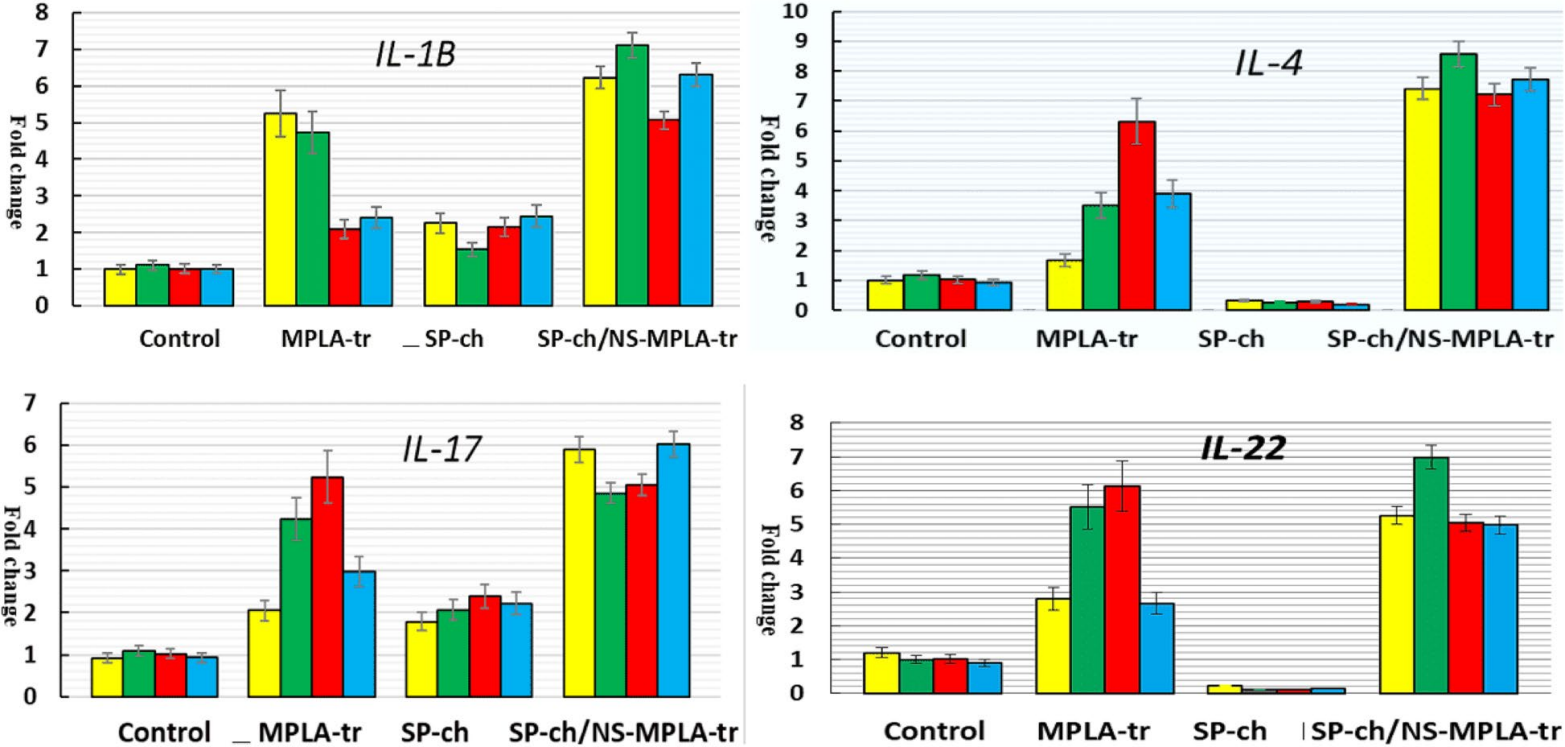
Relative-fold of *IL-1B, IL-4, IL-17*, and *IL-22* genes expression in the four chick Group I: Control; Group II: NS-MPLA supplied (MPLA-tr); Group III: *Salmonella pullorum*-challenged (SP-ch); Group IV: *Salmonella pullorum* challenged and then NS-MPLA-treated (SP-ch/NS-MPLA-tr) day 1 (yellow) day 2 (green) day 3 (red) day 7 (blue)

**Fig. 7:**
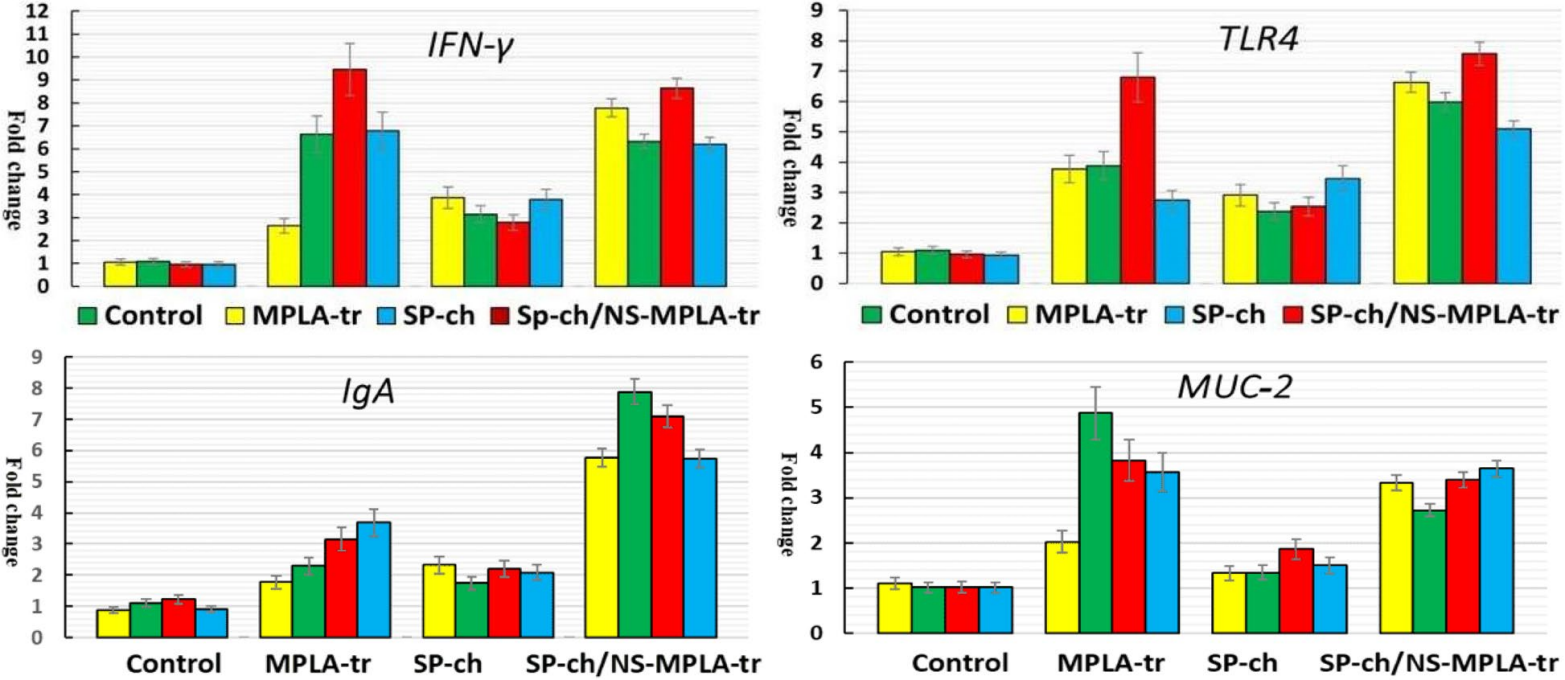
Relative-fold of *IFN-γ, TLR-4, IgA*, and *MUC-2* genes expression in the four chick groups at different time intervals. Group I: control; Group II: NS-MPLA supplied (MPLA-tr); Group III: *Salmonella pullorum*-challenged (SP-ch); Group IV: *Salmonella pullorum* challenged and then NS-MPLA-treated (SP-ch/NS-MPLA-tr) day 1 (yellow) day 2 (green) day 3 (red) day 7 (blue)

The transcriptional regulation of mRNA encoding *IgA, INF-γ, TLR-4, IL-1β, IL-4, IL-17, IL-22*, and *MUC-2* was examined in the four chick groups at the predefined time intervals (days 1–3 and day 7). Comparing the NS-MPLA-supplied, SP-challenged and NS-MPLA-treated chicks (groups II–IV) to the control (group I), differences in the tested gene expression were observed (Figs. 6 and 7). The *MUC-2* and *IgA* gene expressions showed a little rise in the chicks (group III) those were SP-challenged. Significant changes in the expression levels of *IgA, INF-γ TLR-4, IL-1β, IL-17*, and *MUC-2* were observed in the same group; these changes ranged from two to three times that of the control group, with a sharp decline in the expression levels of *IL-4* and *IL-22* (Fig. 6). The chicks in group II, who had only received the NS-MPLA, were different from those in group I in terms of the mRNA relative fold increases in the investigated gene expression, as shown in Figs. 6 and 7. The expression of the targeted gene displayed a discernible pattern in the chicks that SP challenged and then NS-MPLA treated (Group IV). All genes examined exhibited a 5- to tenfold shift at the predefined intervals following the chicks' challenge and subsequent NS-MPLA treatment (Figs. 6 and 7). Chicks in group IV exhibited the highest gene expression, particularly when compared to the SP-challenged chicks.

## Discussion

In *vitro* and in *vivo,* studies on several animal species have demonstrated the potent immunomodulatory and antibacterial activities of herbal plant derivatives. Infected animals are successfully cured when *N. sativa* is used as an antibiotic [42, 43]. The immunomodulatory activity increases immunity and generates non-specific cellular protective immunological responses on the gastrointestinal tract's mucosal surface [44, 45]. For the first time in veterinary medicine, novel herbal and microbial-derived compounds enclosed as NS-MPLA nanoliposomes were developed and experimentally tested against virulent PDR-SP that were previously isolated from infected broiler chicks. When orally administered, NS-MPLA nanoliposomes were able to modulate the immune-related cytokines and mucosal regeneration-associated gene expression, drastically reduce the clinical signs, enhance lymphocytes and innate immune cell trafficking in the internal organs, and stop SP fecal shedding and liver tissue's colonization in the tested broiler chicks. Until recently, one study [43] used *N. sativa* oil chitosan nanoparticles as immunomodulators in broiler chicks infected with Salmonella Enteritidis and another by [16], who used *N. sativa* in crude form with silver oxide nanoparticles against coccidiosis in commercial poultry.

The need for further research is highlighted by the fact that, despite the use of chicken models in studies on many diseases, nothing is known about how to protect and/or treat chickens from Salmonella infection during their early growth stages. Here, a strain of *S. pullorum* (SP) that has been previously isolated from clinically infected chickens was administered to early-aged broiler chicks. The SP strain, when tested against nine antimicrobials from different antibiotic families, was shown to be resistant to them (pandemic-drug-resistant, or PDR). Additionally, the strain showed resistance to the potent antibacterial agent, pure *N. sativa* essential oil, indicating that the antimicrobials were unable to stop a Salmonella infection and proving the need for an alternative. There have been prior reports of virulent, multidrug-resistant isolates of Salmonella species [46, 47]. The reported bacterial resistance indicates the strict need for antibiotic legislation and planning protocols limiting their abuse or finding an alternative protocol to avoid the emergence and spread of those microbes in the community.

The immunomodulatory and therapeutic properties of NS-MPLA nanoliposomes that were developed and investigated in this work. TEM and Zeta potential investigations verified that the chitosan-encapsulated NS-MPLA was successfully formed, with an average size in the liposomal bilayer ranging from 10 to 45 nm. There have been reports of hydrophobic compounds being encapsulated in liposomal bilayers [48, 49]. The outer chitosan layer and its well-known cationic characteristics were linked to the produced nanoliposomes' positive zeta potential (+ 8.24) [43]. The tiny, negatively charged peaks could be associated with unreacted MPLA or NS molecules. The results of the investigation indicated that one of the factors contributing to the immunotherapeutic efficacy of NS-MPLA was the size of the created nanoliposomes (> 10 nm). Immunogens encapsulated in > 2 nm nanoparticle sizes have been shown to be more biodegradable, biocompatible, less toxic, and readily producible, which makes them potentially effective in medication efficacy and distribution, as previously recorded [50–52]. Conversely, the majority of intestinal regions absorb those encapsulated immunogens in less than 2 nm, which results in feeble, age-dependent immune responses [50]. Because of their positively charged surfaces, larger nanoparticles (> 10 nm) effectively encapsulate the immunogens, aid in attachment to host cells, and delay the release of the antigen for extended periods of time, all of which contribute to a steady immune response.

Clinical symptoms developed two to three days after the oral challenge with SP) and they progressively worsened over time. The development of pathological lesions in the internal organs, bacterial shedding and colonization of the liver tissues, and clinical symptoms all supported the SP infection. The signs of the SP infection were similar to those of Salmonella Typhimurium infection [53] and those observed in chicks infected with Salmonella Pullorum/ Gallinarum [54]. The virulence of the SP strain with effective colonization is confirmed by the gross lesions, which comprised catarrhal enteritis, congestion, and hemorrhages throughout the digestive tract in early-aged chicks [55]. Congestion and hemorrhages in the lungs, spleen, and gut of the infected chicks were all documented, as observed in our study [56, 57]. The intestinal flora, the type and dosages of the infectious microbial strain, or the immaturity of the systemic and mucosal immune systems at early ages could all be contributing factors to the infection [45, 53–58], varying mortality rates in chicks infected with SP and/or other Salmonellae species have been documented, indicating economic losses and the requirement for a protective therapeutic control program against Salmonella infection.

*N. sativa* oil and/or seed components have been reported to have antibacterial activity against different pathogens and have immunomodulatory effects in chickens and other species both in *vitro* and in *vivo* [9, 10, 12, 44, 59]. In order to achieve the desired efficacy on the targeted parameters, the researchers of those studies used *N. sativa* as a dietary inclusion of multipurpose feed additives at various doses for a prolonged period of time in the form of seeds or crude oil. The data from those studies included a number of negative aspects, such as the use of different crude preparations of *N. sativa* seeds or oil at different concentrations (10–20%) without taking age or costs into account. The controversy in those studies could be attributed to the variable ingredients in *N. sativa* oil, such as volatile oils, saponins, alkaloids, and other antinutritional elements. In a study by [12], they examined the efficacy of diets supplemented with oil or seed (1–5, and 10 or 50 g/kg) on broilers' growth performance and immunity and noted no positive impact on broilers’ growth performance or immunity. The possible causes could be interference with the protective immune response or the suppressive activity of the antinutrient bioactive component concentrations used, which may affect both pathogenic and beneficial commensal microbes. The inconsistencies among the studies could also be due to differences in basal diet formulations, chicken race and age, the virulence of microbial strains, inoculation time, duration of the study, and the quality and quantity of active components in the *N. sativa* seed or extracted oil.

The use of natural herbal products, such as *N. sativa* purified oil, together with a well-known immunomodulatory adjuvant such as MPLA, could be an easy-to-administer solution for protection against microbial infection as well as a safe antimicrobial immunomodulator. Applying NS-MPLA nanoliposomes as antimicrobial therapy and as immunomodulators to the chicks that were infected with SP resulted in the resolution of the clinical symptoms and curing organ lesions with bacterial colonization prevention, as demonstrated by the current study results. The recovery and alleviation of organ lesions in the NS-MPLA-treated chicks could be attributed to three factors: (i) the coordination of the different gut components to repair the damaged tissues as a result of MUC-2 regulation; (ii) the immune cells and lymphocytic aggregates localizing to the internal organs; and (iii) the modulation of the innate and specific immune responses as a result of immune-related gene stimulation.

For the gut to function effectively, the specialized aspects of the intestinal components must be coordinated. When compared to the chicks that were SP challenged and not treated with NS-MPLA, notable intestinal tissue repair was observed after the chicks were treated with NS-MPLA post-SP challenge. The observed intestinal and internal organ lesions repair could be attributed to the following: i) the coordination of the major gut components (enterocytes goblet cells, localized immune cells, and the mucous layer) under the effect of NS-MPLA treatment; ii) improved biodegradability and bioavailability [50, 51] of the NS-MPLA nanoliposomes; iii) the noted trafficking of macrophage cells to internal tissues, which are known for their capacity to reconstruct damaged tissues; iv) the modulation of MUC-2 gene-expression and its downstream production of cell-building and mucous layer protective proteins by epithelial cells [60–62]; v) upregulation of mucosal IgA with the immune-related cytokines genes known for their microbial infection control and protection against the release of harmful microbial components; and vi) enhancement of the mucosal microbial recognition markers, such as TLR4 [63]. Those could be explanatory causes for the rapid intestinal tissue repair seen in the SP-challenged and then NS-MPLA-treated chicks. They could also be reasons behind the colonization-limiting and immunity-boosting immunity of the treated chicks against the harmful effects of SP-induced infection.

*N. sativa* essential oil (NS) has been reported to have antimicrobial properties and increase animals' IgA, IgM, and IgG levels [64, 65]. As aforementioned of this discussion, the dose, growth stage, and nature of the used *N. sativa* preparations are major drawbacks of these studies. Combining NS with MPLA, which is well-known for its immunostimulant properties [21] [26, 27], in nanoliposomal form, NS-MPLA, produced protection against salmonella infection in broiler chicks. Due to a lack of research studies related to MPLA in the poultry industry and immunity, we relied on human and mouse models to explain our study results. The detected gene overexpression may be attributed to the synergistic activity of the immunostimulatory MPLA [22–24] and the high concentrations of phenolic and other antioxidant compounds contained in the NS purified oil extract that are known for their strong immunostimulatory activities, including immunoglobulin secretion and cytokine modulation [11] [66–68]. The immune-related mRNA expression in the cecal tissues may be a result of immune cell tissue infiltration and aggregation and could explain rapid clinical recovery, healing of organ lesions, and microbial elimination from the liver and fecal droppings. Our results agree with [69], who found that NS influences the splenocyte secretion of Th2 cytokines responsible for humoral immune responses in a dose-dependent manner, and with [70], who demonstrated that a methanolic extract from NS stimulated the expression of inflammatory genes coding the humoral and cellular cytokines *IL-1β, IL-4*, and *IFN-γ*. However, those studies were performed in *vitro* using NS as a crude extract in dose-dependent manners. As indicated in the results of our study, with NS-MPLA administration, IgA expression levels significantly increased at the different time intervals. The high fold change in IgA gene expression in the chicks treated with NS-MPLA post-SP challenge may be due to the trafficking of specific immune cell recruitment and damaged tissue repair noticed, as similarly in regard to *IL-1, IFN-γ*, and *TLR-4* previously reported [43] after using chitosan-*N. sativa* nanoparticles in broiler chicks challenged with Salmonella Enteritidis.

One of the study's intriguing conclusions is that, in contrast to the other experimental chick groups, which displayed an increase in the expression levels of the same genes, the SP-challenged chicks showed a significant decrease in IL-4 and IL-22 gene expression and a slight increase in IL-17 gene expression. The challenged chicks showed limited immune cell organ localization, tissue damage, and lymphoid tissue depletion in addition to the markedly decreased IL-4 and IL-22 mRNAs. These factors might influence Th1/Th2 responses and could be novel ways for Salmonella to evade immune responses that are meant to protect it. Studies of IL-17 and IL-22 in birds are rare, despite the fact that these molecules have been investigated in other species. A prior study [71] demonstrated that the *N. sativa* extract had a stimulatory effect on Th1 cells and an inhibitory effect on Th2 cells by increasing the production of IFN-γ while inhibiting IL-4. Primarily, IL-17 is a pro-inflammatory cytokine that facilitates granulopoiesis, neutrophil accumulation in peripheral tissues, and the release of antimicrobial peptides from epithelial cells, all of which aid in coordinating defense against infections [72]. However, IL-22 seldom contributes to inflammation and is responsible for preserving the integrity of internal organs and tissues through homeostatic cytokine production [73, 74]. Innate immune cell organ localization, lymphocytes, and the development of cellular Th1 responses are the main factors associated with the expression of IL-17 and IL-22. Both the restricted immune cell trafficking to the internal organs of the SP-challenged chicks and the mild production of IL-17 may result in damages to the villi and the lining epithelium [73, 74]. The development of protective immunity was demonstrated by the NS-MPLA-treated chicks' observable recovery of clinical symptoms, remission of internal organ lesions, and immune-related gene expression modulation following the SP challenges in this experimental trial. These findings may point to NS-MPLAs as a promising therapeutic agent against SP infection.

Our research is prioritized over the others' previous research. The MPLA, which has been previously found to be safe and to demonstrate strong immunomodulation in clinical trials on both mouse and human models, is being employed for the first time in poultry research in combination with NS (NS-MPLA) for immunity and protection against SP. Based on the study's findings, we conclude that the NS-MPLA nanoliposome can be applied to chick protection programs for those reasons: i) There was an evident increase in genes linked to immune cytokines, mucosal tissues, and IgA production that promote intestinal repair and prevent bacterial colonization; ii) the SP clinical symptoms resolved quickly following the oral dosage, potentially avoiding antibiotic-related costs; iii) the internal organs (intestine and liver) were completely free of SP within three to four days after an SP challenge; iii) the genes for mucosal surfaces, immune-related cytokines, and IgA that promote intestinal repair and prevent bacterial colonization, as well as the development of effective innate and specific immune responses, are significantly increased. NS-MPLA can provide protection against other intestinal infections, including *Enterobacteriales*, because lipid A is a necessary component shared by all *Enterobacteriales* members. One can increase immunity, extend protection against different intestinal lipopolysaccharide-containing bacteria, and avoid the costs of treating each bacteria separately using NS-MPLA. The immunoprotective ability of the NS-MPLA against Salmonella infection and carrier states in laying chickens need to be investigated.

## Supplementary Information

Supplementary Material 1.

## Data Availability

All the data are provided in the manuscript.
