# Supplementary material for: Nigella sativa monophosphoryl lipid A nanoliposome: a promising antibiotic alternative and immunomodulator to control virulent pandemic drug-resistant Salmonella pullorum infection in broiler chicks
Source: BMC Vet Res. 2025 Mar 3;21:132. doi: 10.1186/s12917-025-04473-w (PMC11874670; doi:10.1186/s12917-025-04473-w)
Supplement: Supplementary file 1 — Supplementary Material 1. [file 12917_2025_4473_MOESM1_ESM.pptx]

## Slide 1
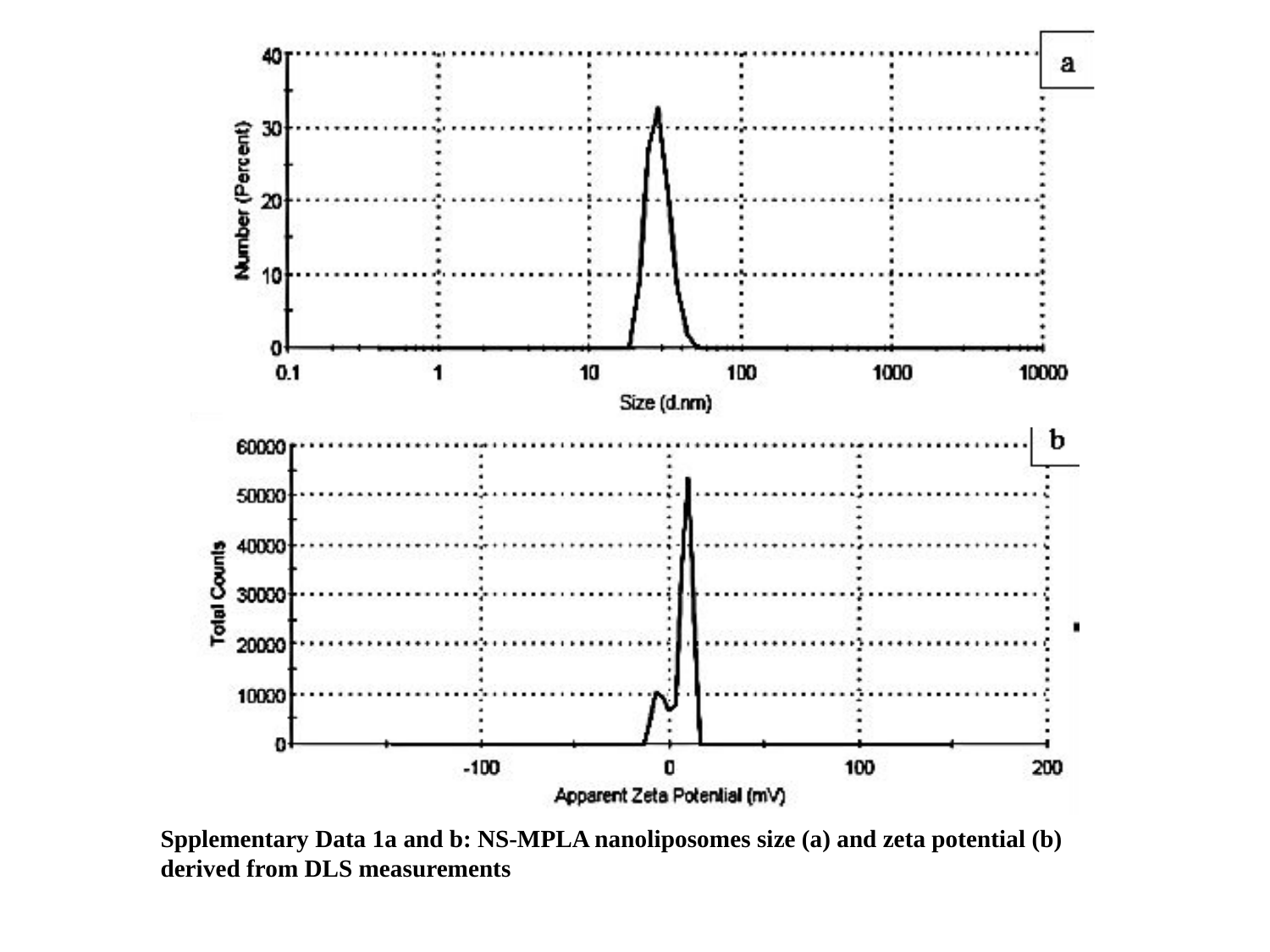

Spplementary Data 1a and b: NS-MPLA nanoliposomes size (a) and zeta potential (b) derived from DLS measurements

## Slide 2
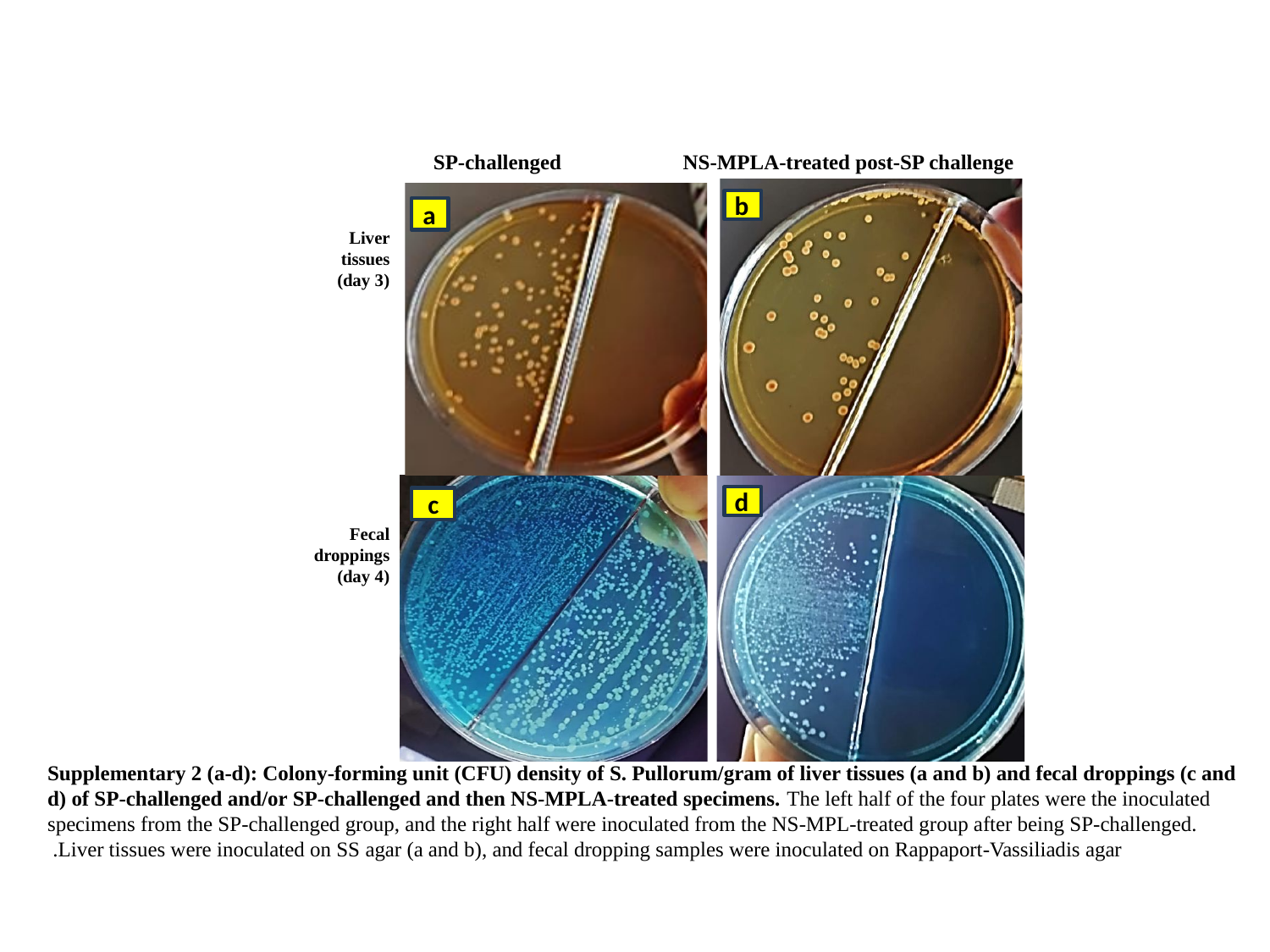

SP-challenged NS-MPLA-treated post-SP challenge
b
a
d
c
Liver tissues
(day 3)
Fecal droppings
(day 4)
Supplementary 2 (a-d): Colony-forming unit (CFU) density of S. Pullorum/gram of liver tissues (a and b) and fecal droppings (c and d) of SP-challenged and/or SP-challenged and then NS-MPLA-treated specimens. The left half of the four plates were the inoculated specimens from the SP-challenged group, and the right half were inoculated from the NS-MPL-treated group after being SP-challenged. Liver tissues were inoculated on SS agar (a and b), and fecal dropping samples were inoculated on Rappaport-Vassiliadis agar.
